# Evaluation of PhageDX *Salmonella* Assay for *Salmonella* Detection in Hydroponic Curly Lettuce

**DOI:** 10.3390/foods10081795

**Published:** 2021-08-03

**Authors:** Nathanyelle Soraya Martins de Aquino, Susana de Oliveira Elias, Eduardo Cesar Tondo

**Affiliations:** Laboratório de Microbiologia e Controle de Alimentos, Instituto de Ciência e Tecnologia de Alimentos, Universidade Federal do Rio Grande do Sul Campus do Vale-Agronomia (ICTA/UFRGS), Av. Bento Gonçalves 9500, Porto Alegre 91501-970, RS CEP, Brazil; susanaelias@gmail.com (S.d.O.E.); tondo@ufrgs.br (E.C.T.)

**Keywords:** bacteriophage, diagnostics, leafy green

## Abstract

Lettuce is one of the most consumed leafy vegetables worldwide and has been involved in multiple foodborne outbreaks. *Salmonella* is one of the most prevalent etiological agents of foodborne disease (FBD) in lettuces, and its detection may take several days depending on the chosen method. This study evaluates a new rapid method that uses recombinant bacteriophages to detect *Salmonella* in hydroponic curly lettuce. First, the ability of the assay to detect six *Salmonella* serovars at three different concentrations (1, 10, and 100 CFU/well) was tested. Second, the detection of *Salmonella* was tested in lettuces using a cocktail of the same *Salmonella* serovars and concentrations after a 7 h enrichment. The results of these experiments showed that the detection limit was dependent on the serovar tested. Most serovars were detected in only 2 h when the concentration was 100 CFU/well. *Salmonella* was detected in 9 h (7 h enrichment + 2 h bioluminescence assay) in all lettuce samples with 10 CFU/25 g or more. *Salmonella* detection was not influenced by natural microbiota of lettuces. This study demonstrated that the phage assay was sensitive and faster than other detection methods, indicating that it is a better alternative for *Salmonella* detection on lettuces.

## 1. Introduction

Recently, the consumption of fresh-vegetable salads has gained popularity worldwide due to the high concentration of bioactive compounds such as vitamins, minerals, and antioxidants, which are important for human health [1,2]. This rapid increase resulted in consumption growth rates of 10–20% per year [3,4]. However, reports on foodborne disease (FBD) outbreaks have multiplied, many of them caused by pathogens present on fresh vegetables and salads [5]. Therefore, the microbiological safety of fresh produce has become an important public health issue. Contamination may occur at any point along the production chain [6], and there are no thermal processing steps or sanitization procedures able to completely inactivate all possible pathogens in fresh vegetables before consumption.

Lettuce is the most produced and consumed leafy vegetable crop in the world. From 1980 to 2016, it was the vegetable most implicated in FBD in developed countries with *Salmonella* being the main culprit [6,7]. This pathogen is one of the most common causes of FBD worldwide and is responsible for severe economic losses, considering bacterial pathogens [8]. The *Salmonella* genus is composed of over 2700 serovars, of which 200 are commonly associated with human salmonellosis. Of these, *S.* Typhimurium and *S*. Enteritidis are the most frequently implicated in human salmonellosis [7].

The current gold standard method for *Salmonella* detection needs at least three days for a negative result due to multiple sample enrichments and plating on selective agars. In cases where a presumptive *Salmonella* colony is found, additional steps are required to confirm its identity [9]. Nevertheless, traditional methods can detect 1 CFU of *Salmonella* in a 25 g sample which is the current acceptable limit for several foods in diverse microbiological regulations. Other assays using ELISA, latex agglutination, PCR, mass spectrometry, and metagenomic sequencing have been developed with the aim of reducing the time needed for food pathogen detection. These approaches have been successful; however, most available methods still require at least 18 h of enrichment to detect 1 CFU/25 g [10]. In addition, many of these methods could potentially detect non-viable *Salmonella* cells, resulting in the need for further confirmation testing using traditional methods.

Recently, bacteriophages have been used to detect bacterial foodborne pathogens due to their safety, specificity, rapid propagation, and ability to differentiate between live and dead cells [11]. The ability to differentiate living cells and dead cells is an advantage over many rapid methods. Rapid propagation is an advantage over traditional methodology. The high specificity of bacteriophages, eliminates the need for isolation steps of the target pathogen, as used in traditional methodologies, and this decreases the total assay time. One promising phage-based approach for *Salmonella* detection is the use of recombinant phages that carry a luciferase reporter. NanoLuc^®^ is an engineered luciferase from a deep-sea shrimp *Oplophorus gracilirostris* that is 150 times brighter than other luciferases and reacts with a novel furimazine substrate with low background noise [12]. Based on these characteristics, NanoLuc^®^ would be a superior choice as a luciferase reporter in phage-based assays. PhageDx *Salmonella* Assay is a new method that uses recombinant bacteriophages with NanoLuc^®^ inserted to detect *Salmonella*. This method detected several Salmonella serovars in in vitro conditions, showing limits of 10–100 CFU detection per mL (without enrichment). Besides this, the assay detected 1 CFU in either 25 g of ground turkey with a 7 h enrichment or 100 g of powdered infant formula with a 16 h enrichment. However, the PhageDx *Salmonella* Assay has not been tested with lettuces [12]. The objective of this study is to assess the performance of the PhageDx *Salmonella* Assay for the detection of Brazilian *Salmonella* strains in vitro and on hydroponic curly lettuce.

## 2. Materials and Methods

### 2.1. PhageDx Salmonella Assay

The method used in this study was developed by the Laboratory Corporation of America (LabCorp) and registered in AOAC^®^ (Certificate No. 121904). The method has been described in detail in Nguyen et al. [12]. Briefly, the assay contains two recombinant bacteriophages, SEA1.NL and TSP1.NL which have had the NanoLuc^®^ (Promega Corp., Madison, WI, USA) gene inserted in their genome by homologous recombination. The test is based on the infection of recombinant-bacteriophages in *Salmonella* spp. cells, resulting in the production of the NanoLuc^®^ luciferase during phage replication. After a 2 h infection, luciferase substrate is added, and the sample is read on a luminometer. Readings above a pre-established cutoff of 750 relative light units (RLU) indicate the presence of *Salmonella*, and readings ≤750 RLU indicate absence of *Salmonella*. The bioluminescence assay is further detailed in Section 2.4.

### 2.2. In Vitro Assay for Determination of Detection Limit

Six *Salmonella* serovars were used to test the limit of detection (LOD) of the assay; *S.* Enteritidis, *S*. Typhimurium, *S*. Infantis, *S*. Minnesota, *S*. Heidelberg, and *S*. Saint Paul. All serovars were isolated from foods in Brazil and are from the Laboratory of Food Microbiology and Food Control of Institute of Food Science and Food Technology of the Federal University of Rio Grande do Sul (ICTA/UFRGS). The strains were cultivated overnight in 5 mL of Tryptic Soy Broth (TSB, Kasvi, Brazil) at 37 °C. Using a microplate reader (Loccus LMR 96, Cotia, Brazil), the OD_630_ was determined and then diluted to an OD_630_ = 0.2 (or approximately 10^8^ CFU/mL). Then, 1 mL of each culture was added to a 15 mL conical tube in order to form a pooled culture of *Salmonella*. This condition was tested to simulate a food matrix contaminated by several serovars. The *Salmonella* cocktail and a culture of each individual strain were serially diluted in TSB (Kasvi, Brazil) to concentrations of 1000, 100, and 10 CFU/mL. To determine the level of detection (LOD) of the PhageDx assay, 100 µL of each diluted sample were transferred to a 96-well plate, resulting in 100, 10, and 1 CFU/well. The cell concentrations were confirmed by plating each final cell suspension on Tryptic Soy Agar (TSA, Kasvi, Brazil), incubating at 37 °C for 24 h, and counting colony formation. The bioluminescence test was performed as described in Section 2.4.

### 2.3. Salmonella Detection on Hydroponic Curly Lettuce

The *Salmonella* cocktail was used to inoculate lettuce samples. Before inoculation, 15 mL of the cocktail was centrifuged at 4 °C, for 10 min at 2810× *g* (CIENTEC CT-5000R, Belo Horizonte, Brazil), and the supernatant was discarded. Then, the pellet was washed three times with sterile 0.1% Peptone Water (Kasvi, Brazil). After the final wash, cells were suspended in sterile 0.1% Peptone Water (Kasvi, Brazil). A microplate reader was used to determine OD_630_, and cells were diluted to a concentration of approximately 10^8^ CFU/mL (OD_630_ = 0.2). Next, the *Salmonella* cocktail was serially diluted with sterile 0.1% Peptone Water (Kasvi, Brazil) to final concentrations of 100, 10, and 1 CFU/mL. Cell concentrations were confirmed by plating on TSA (Kasvi, Brazil) as described above.

Hydroponic curly lettuce was purchased at a hypermarket in Porto Alegre (Brazil) and transported to the Laboratory of Food Microbiology and Food Control located at the Federal University of Rio Grande do Sul (ICTA/UFRGS). The lettuce, previously tested for the absence of *Salmonella* spp., was portioned in 25 g samples and individually placed inside Whirl-Pak^®^ sterile filter bags (Nasco, Fort Atkinson, WI, USA). Lettuce samples were artificially contaminated by inoculating onto the leaf surface with 1 mL of 100, 10, or 1 CFU/mL *Salmonella* cocktail dilutions. The *Salmonella* final concentration on lettuce were 100, 10, 1 CFU/25 g. Then, 75 mL of pre-warmed (41 ± 1 °C) Buffered Peptone Water (BPW, Merck, Darmstadt, Germany) were added, and the samples were homogenized using a stomacher (Stomacher^®^ 400, Seward, England) for 30 s. Finally, all the samples were incubated at 41 ± 1 °C for 7 h, and the bioluminescence assay was carried out as described in Section 2.4.

### 2.4. Bioluminescence Assay

The bioluminescence assay was performed using 100 and 150 µL of samples prepared according to Section 2.2 and Section 2.3, respectively. Four or 10 replicates of each dilution were transferred to a 96-well white plate (Thermo Scientific™, Waltham, MA, USA). Samples included 4 × 10^2^ CFU/mL, 10 × 10^1^ and 10 × 10^0^ CFU/mL samples. A total of 10 µL of the recombinant phage cocktail from the PhageDx *Salmonella* Assay were added to each well. The samples were incubated for 2 h at 37 °C. The luciferase reagent mix was prepared by combining 50 µL of NanoGlo buffer, 1 µL NanoGlo substrate (Nano-Glo^®^ Luciferase Assay System, Promega Corp., Madison, WI, USA), and 15 µL Renilla lysis buffer (Renilla Luciferase Assay System, Promega Corp., Madison, WI, USA). After infection, 65 µL of the luciferase reagent mix was added to each well, and the 96-well plate was read immediately in a GloMax^®^ Navigator Luminometer (Promega, Fitchburg, MA, USA). The reading parameters used were 3 min wait time, 1 s integration, and two reads for a plate. Relative light units (RLU) were the signal output. All assays were performed in triplicate. In total, each *Salmonella* serovar and the cocktail were tested 30 times for low and medium inoculum and 12 times for the high inoculum. The results were expressed as the percentage of positive tests. The means, standard deviations, and coefficient of variation were calculated using Excel^®^ version 2016 (Microsoft Co., Ltd. Redmond, WA, USA).

In the in vitro assay, the negative controls consisted of uninoculated TSB culture medium. The negative control of the food test was uninoculated lettuce sample added to BPW (Merck, Darmstadt, Germany).

## 3. Results

Table 1 shows the RLUs generated by the PhageDx *Salmonella* Assay in culture tests and artificially contaminated lettuce samples. The average RLUs of the in vitro samples ranged from 182 to 26,543, while the RLUs generated by lettuces ranged from 1914 to 106,579. As expected, RLUs increased according to the size of the inoculum (Table 1). *S.* Typhimurium showed the highest RLU values even with the lowest inoculum, followed by the *Salmonella* cocktail.

Table 2 contains the percentages of detection of each *Salmonella* serovar, and *Salmonella* cocktail evaluated in vitro, and the results of *Salmonella* cocktail on lettuce samples. The percentages of detection varied from 0% (when the signals emitted by samples were less than 750 RLU) to 100% (when all samples emitted signals above 750 RLU). As expected, the lowest percentages of detection were those from samples with low inoculum, while the detection percentages increased according increased the inoculum size. In in vitro tests, 100% of *S.* Saintpaul, *S.* Infantis, *S.* Heidelberg, *S.* Typhimurium, and the cocktail of *Salmonella* were detected without pre-incubation just 2 h of infection at 100 CFU/well. S. Minnesota and *S.* Enteritidis could also be identified at the highest inoculum concentration, but had lower detection rates, 75% and 50%, respectively. Table 2 also demonstrated that 100% of artificially contaminated lettuces presenting 10 and 100 CFU per 25 g were also detected.

## 4. Discussion

Since 1987, the American Public Health Association (APHA) has pointed out that rapidity and sensitivity are two critical requirements for pathogen detection methods used in food industries. Rapid detection is essential because food industries need to know as quickly as possible whether or not pathogens are present in final products. The sensitivity is important because legal requirements generally require the absence of pathogens like *Salmonella* in 25 g of food because the infective doses of these pathogens can be as low as a single cell [13]. In addition, pathogens can multiply due to time and temperature abuses during food production, increasing the risk of foodborne illnesses.

PhageDx *Salmonella* Assay was developed to meet the criteria of rapidity and sensitivity to be used in food industries worldwide. The results of this study demonstrated that assay was able to detect *Salmonella* isolated in Brazil when present on lettuces containing natural microbiota. Lettuce was chosen as a food matrix to be tested because it is one of the most consumed leafy greens worldwide and is frequently linked with foodborne salmonellosis. Besides, the PhageDx *Salmonella* Assay was not tested with this food matrix during its recent development [12]. Our results demonstrated that the PhageDx *Salmonella* Assay detected all serovars evaluated, isolated from animal sources in Brazil (from foods involved with FBD and from poultry carcasses), generating a range of RLUs (Table 1). This was consistent with Nguyen et al. [12] findings that also demonstrated that different RLU counts were obtained from different *Salmonella* serovars. For example, 52,329.1 RLU for *S.* Minnesota USDA; 419,753,056 for *S.* Enteritidis 1294; 1,218,853,760 RLU for *S.* Saintpaul SARB55; 7549 RLU for *S.* Infantis JUL 301; 207,102,224 RLU for *S.* Heidelberg 6316-J. These RLU values were obtained after 2 h of phage infection in stationary phase *Salmonella* (concentration of approximately 10^8^ CFU/mL) with the PhageDx *Salmonella* phage reagent. The same authors also found that the same *Salmonella* serovar can produce different RLU numbers. These results can be explained because the RLU numbers are dependent on the bacterial multiplication rate during the pre-enrichment period. Some strains grow more slowly than others, resulting in fewer phages and, consequently, lower RLU signals. In in vitro experiments, there was no enrichment step of the samples, which could allow the increase of *Salmonella* numbers, increasing RLUs as well.

The previously defined criteria [12] considered positive all those whose RLU signal emitted was greater than or equal to 750 RLU, which justifies the increase in the standard deviation and the variation coefficient with the increase in the number of *Salmonella* cells. This is because, in the same set of samples with the same inoculum concentration, we obtained positive samples with RLU from 831 to 4051 (data not shown). Moreover, all these samples are considered positive as they meet the criteria of ≥750 RLU.

As demonstrated in the results of Table 2, most samples contaminated with *Salmonella* serovars were 100% positive when the level of 100 CFU was used. The differences observed in the phage’s ability to detect different serovars can be explained by factors other than the rate of bacterial growth. For example, *S*. Typhimurium showed a high percentage of detection even when the inoculum was as low as approximately 1 CFU/well. The adhesion stage between bacteriophages and target cells is the first step towards the success of the infection process, both in nature and in phage assays. In this stage, the bacteriophages will adhere to structures present on target cells, called receptors. These receptors can be proteins, lipopolysaccharides, teichoic acids, and capsules [14]. Due to mechanisms that have not yet been fully explained, the same phage can bind to several receptors on a target cell, facilitating the adhesion process [15]. However, changes in the structures of these receptors can totally or partially compromise the adhesion of bacteriophages [16]. As we observed in the present study, the phage recognition of different *Salmonella* serovars was not compromised. Although all strains tested positive at the high level of 100 CFU, only *S*. Typhimurium and *S.* Heidelberg were positive at all levels, 1, 10 and 100 CFUs. The other strains tested required 100 CFU before testing positive. The detection capacity of different *Salmonella* species by the phages SEA1.NL and TSP1.NL, which compose PhageDx *Salmonella* Assay, was previously demonstrated [12]. Similar to our results, different serovars also emitted different amounts of RLUs and did not compromise the detection effectiveness of the test.

The PhageDx *Salmonella* Assay detected different *Salmonella* serovars in different concentrations in vitro. Then, we tested the *Salmonella* detection on food. Leafy vegetables eaten raw are known to be important carriers of human pathogens [17]. Lettuce has proved to be one of the most important foods for spreading FBD outbreaks in developed countries [7]. In the present study, we observed that contaminated lettuce samples incubated for 7 h before the phage infection generated higher numbers of RLUs when compared to the in vitro tests where there is no enrichment time (Table 1). These results can be explained because the number of *Salmonella* cells during the phage infection phase was higher, increasing RLU signals.

Considering the smallest inoculum (approximately 1 CFU/mL), 30% of lettuce samples were positive for *Salmonella* (Table 2). *Salmonella* counts carried out on agar plates revealed that the actual amounts of *Salmonella* inoculated on the lettuces ranged from 1.7 to 3.3 CFU/25 g in the low concentration. Considering the use of such a low number of cells as inoculum of a specific food matrix, one cannot discard the possibility that some samples were not inoculated by any pathogen cell [18], explaining the negative results obtained in some samples. Beyond that, we observed that this slight variation within the same inoculation range could vary the detection rate (data not shown). However, we do not know if it is because there were no cells to detect in the sample or because of the assay’s real detection limit. When the inoculum was increased by 10 × and 100 ×, the detection percentages reached 100%.

The averages of mesophilic microorganisms and total coliform on lettuces were 6 and 4 log CFU/g, respectively (data not shown). These values are in accordance to previous studies carried out in Brazil, which demonstrated that total bacterial count ranged from 4 to 7 log CFU/g on lettuces or lettuce salads [19,20], and coliforms count ranged from 3.11 to 4.69 log CFU/g on conventional and organic lettuce [21]. The assay detected low counts of *Salmonella* even with high amounts of mesophilic microorganisms and total coliforms on lettuce, indicating that natural microbiota did not compromise the *Salmonella* detection. One of the advantages of using bacteriophages to detect pathogens is the specificity and this was demonstrated by these results. The genetic similarity between *Salmonella*, the target microorganism, and microorganisms belonging to the coliform group did not affect the test’s specificity. Furthermore, there were no false positives in the assays. All lettuce samples with their natural microbiota that were not inoculated with *Salmonella* were negative.

Ultimately, PhageDx *Salmonella* Assay, in addition to being rapid and sensitive, does not require additional technologies to detect the target microorganism. When compared to other recently published detection methods using bacteriophages, such as phage amplification combined with qPCR [22], phagomagnetic separation with enzymatic colorimetry [23], and bacteriophage amplification coupled with mass spectrometry [24], this method was simple to perform and required little equipment to carry out the assays.

In an approach similar to the one used in this work, using recombinant bacteriophages, the assay’s detection limit was approximately 400 CFU/25 g of ground beef; 10 CFU/cm^2^ in romaine lettuce [25]. Kim et al. [25] used 5 h enrichment time and 40 min of phage infection period.

In conclusion, the method analyzed in this study demonstrated to be sensitive and specific, making it an excellent assay to be used to detect *Salmonella* on lettuces. The assay detected 100% of samples with 10 CFU/25 g, and some samples probably containing 1 CFU/25 g after 7 h of enrichment and 2 h of phage infection. This time is less than those needed by other *Salmonella* detection methods available on the market. In addition, the method was easy to perform, did not require washing or concentration steps, and was not affected by the lettuce matrix or its microbiota. These represent significant advantages over traditional and rapid methods available on the market. More tests should be performed to evaluate the PhageDx *Salmonella* Assay in other food matrices.

## Figures and Tables

**Table 1:** Relative light unit (RLU) numbers due to the detection of *Salmonella* in vitro and on curly hydroponic lettuce by PhageDx *Salmonella* Assay.

Sample	Inoculum	Number of Replicates	Avg. RLU	SD	% CV
*S*. Minnesota	Low	30	187	41	22
	Medium	30	223	89	40
	High	12	1475	953	65
*S*. Enteritidis	Low	30	182	45	25
	Medium	30	246	168	68
	High	12	724	339	47
*S*. Saintpaul	Low	30	198	115	58
	Medium	30	407	398	98
	High	12	3388	2061	61
*S*. Infantis	Low	30	214	169	79
	Medium	30	479	415	87
	High	12	1914	1324	69
*S*. Heidelberg	Low	30	325	220	68
	Medium	30	873	426	49
	High	12	6158	1729	28
*S*. Typhimurium	Low	30	1814	1305	72
	Medium	30	14,648	9257	63
	High	12	16,715	15,1623	907
*Salmonella* cocktail	Low	30	2323	339	15
	Medium	30	3054	1559	51
	High	12	26,543	9980	38
Lettuce	Low	30	1914	6813	356
	Medium	30	7347	6207	84
	High	12	106,579	68,315	64

Strains were diluted from log phase cultures, 100 µL samples used to give three levels: low (~1 CFU/well), medium (~10 CFU/well), and high (~100 CFU/well). The samples were infected with the phage cocktail for 2 h at 37 °C. Spiked lettuce samples were enriched for 7 h at 41 ± 1 °C in buffered peptone water (BPW) prior to phage infection step. Luciferase substrate mix was added and RLUs (Relative Light Units) were measured using a luminometer. Averages (Avg. RLU), standard deviations (SD), percent coefficient of variation (% CV) were calculated.

**Table 2:** Percentage of *Salmonella* detection of in vitro test and curly hydroponic lettuces contaminated with the *Salmonella* cocktail.

Samples	Negative Control	Low	Medium	High
*S.* Minnesota	0 (0/2)	0 (0/30)	0 (0/30)	75 (9/12)
*S.* Enteritidis	0 (0/2)	0 (0/30)	6.7 (2/30)	50 (6/12)
*S.* Saintpaul	0 (0/2)	3.0 (1/30)	16.7 (5/30)	100 (12/12)
*S.* Infantis	0 (0/2)	3.0 (1/30)	16.7 (5/30)	100 (12/12)
*S.* Heidelberg	0 (0/2)	10 (3/30)	60 (18/30)	100 (12/12)
*S.* Typhimurium	0 (0/2)	83.3 (25/30)	100 (12/12)	100 (12/12)
*Salmonella* cocktail	0 (0/2)	63 (19/30)	93 (28/30)	100 (12/12)
Lettuces	0 (0/2)	30 (9/30)	100 (12/12)	100 (12/12)

Log phase *Salmonella* cultures were diluted to low (~1 CFU/mL), medium (~10 CFU/mL), and high (~100 CFU/mL) concentrations. Samples were infected with the phage cocktail for 2 h. Spiked lettuce samples were pre-incubated for 7 h in buffered peptone water (BPW) before incubation with bacteriophages. The RLU (Relative Light Unit) of samples were measured using a luminometer.

## References

[1] Hassenberg K., Praeger U., Herppich W.B. (2021). Effect of chlorine dioxide treatment on human pathogens on iceberg lettuce. Foods.

[2] Mir S.A., Shah M.A., Mir M.M. (2017). Microgreens: Production, shelf life and bioactive components. Crit. Rev. Food Sci. Nutr..

[3] Rico D., Martin-Diana A.B., Barat J., Barry-Ryan C. (2007). Extending and measuring the quality of fresh-cut fruit and vegetables: A review. Trends Food Sci. Technol..

[4] Siddiqui M.W., Chakraborty I., Ayala-Zavala J.F., Dhua R.S. (2011). Advances in minimal processing of fruits and vegetables: A review. J. Sci. Ind. Res..

[5] Olaimat A.N., Holley R.A. (2012). Factors influencing the microbial safety of fresh produce: A review. Food Microbiol..

[6] Elias S.O., Noronha T.B., Tondo E.C. (2021). Risk of infection with *Salmonella* and *Escherichia coli* O157: H7 due to consumption of lettuce in southern Brazil. MOJ Food Process. Technol..

[7] Machado-Moreira B., Richards K., Brennan F., Abram F., Burgess C.M. (2019). Microbial contamination of fresh produce: What, where, and how?. Compr. Rev. Food Sci. Food Saf..

[8] Mafi N., Orenstein R., Kuipers E.J. (2018). Salmonellosis. Encyclopedia of Gastroenterology.

[9] Andrews W.H., Wang H., Jacobson A., Hammack T. (2018). BAM Chapter 5: Salmonella. Bacteriological Analytical Manual.

[10] Valderrama W.B., Dudley E.G., Doores S., Cutter C.N. (2016). Commercially available rapid methods for detection of selected foodborne pathogens. Crit. Rev. Food Sci. Nutr..

[11] O’Sullivan L., Bolton D., McAuliffe O., Coffey A. (2019). Bacteriophages in food applications: From foe to friend. Annu. Rev. Food Sci. Technol..

[12] Nguyen M.M., Gil J., Brown M., Tondo E.C., de Aquino N.S.M., Eisenberg M., Erickson S. (2020). Accurate and sensitive detection of *Salmonella* in foods by engineered bacteriophages. Sci. Rep..

[13] Vanderzant C., Splittstoesser D.F., APHA—American Public Health Association (1992). Compendium of Methods for Microbiological Examination of Food.

[14] Heller K.J. (1992). Molecular interaction between bacteriophage and the gram-negative cell envelope. Arch. Microbiol..

[15] Silva J.B., Storms Z., Sauvageau D. (2016). Host receptors for bacteriophage adsorption. FEMS Microbiol. Lett..

[16] Hyman P., Abedon S.T., Laskin A.I., Sariaslani S., Gadd G.M. (2010). Chapter 7: Bacteriophage host range and bacterial resistance. Advances in Applied Microbiology.

[17] Mostafidi M., Sanjabi M.R., Shirkhan F., Zahedi M.T. (2020). A review of recent trends in the development of the microbial safety of fruits and vegetables. Trends Food Sci. Technol..

[18] Hohnadel M., Maumy M., Chollet R. (2018). Development of a micromanipulation method for single cell isolation of prokaryotes and its application in food safety. PLoS ONE.

[19] Saraiva B.M., de Castro Antunes Marques Fernandez E., Fernandez A.T. (2019). Avaliação da eficiência antibacteriana de fermentados acéticos comerciais em saladas de alface (*Lactuca sativa*) comercializadas na cidade de Duque de Caxias, Rio de Janeiro. Vigil. Sanit. Debate Soc. Ciênc. Tecnol. (Health Surveill. Debate Soc. Sci. Technol.) Visa Debate.

[20] Schuh V., Schuh J., Fronza N., Foralosso F.B., Verruck S., Vargas Junior A., da Silveira S.M. (2020). Evaluation of the microbiological quality of minimally processed vegetables. Food Sci. Technol..

[21] Maffei D.F., de Arruda Silveira N.F., Catanozi M.d.P.L.M. (2013). Microbiological quality of organic and conventional vegetables sold in Brazil. Food Control.

[22] Garrido-Maestu A., Fuciños P., Azinheiro S., Carvalho C., Carvalho J., Prado M. (2019). Specific detection of viable *Salmonella* Enteritidis by phage amplification combined with qPCR (PAA-qPCR) in spiked chicken meat sample. Food Control.

[23] Zhang Y., Yan C., Yang H., Yu J., Wei H. (2017). Rapid and selective detection of *E. coli* O157:H7 combining phagomagnetic separation with enzymatic colorimetry. Food Chem..

[24] Rees J.C., Voorhees K.J. (2005). Simultaneous detection of two bacterial pathogens using bacteriophage amplification coupled with matrix-assisted laser desorption/ionization time-of-flight mass spectrometry. Rapid Commun. Mass Spectrom..

[25] Kim J., Kim M., Kim S., Ryu S. (2017). Sensitive detection of viable *Escherichia coli* O157:H7 from foods using a luciferase-reporter phage phiV10lux. Int. J. Food Microbiol..

